# The Periodicity of Yellow Fever

**Published:** 1878-09

**Authors:** 


THE PERIODICITY OF YELLOW FEVER.—The following positions are maintained by Dr. Robert Lawson, in a late number of the *Lancet:*

1. Yellow fever is not a disease always presenting the continued form, but it is met with frequently as a remittent, and sometimes as an intermittent.

2. The sporadic cases, presenting yellowness of surface and black vomit, are also found to have the train of urinary symptoms characterizing yellow fever, and are consequently identical with those met with during an epidemic.—*Ibid.*

